# Psilocybin, a Naturally Occurring Indoleamine Compound, Could Be Useful to Prevent Suicidal Behaviors

**DOI:** 10.3390/ph14121213

**Published:** 2021-11-24

**Authors:** Robertas Strumila, Bénédicte Nobile, Laura Korsakova, Aiste Lengvenyte, Emilie Olie, Jorge Lopez-Castroman, Sébastien Guillaume, Philippe Courtet

**Affiliations:** 1 Department of Urgent and Post Urgent Psychiatry, CHU Montpellier, 34000 Montpellier, France; aistelengvai@gmail.com (A.L.); e-olie@chu-montpellier.fr (E.O.); s-guillaume@chu-montpellier.fr (S.G.); p-courtet@chu-montpellier.fr (P.C.); 2 Institute of Functional Genomics, CNRS, INSERM, University of Montpellier, 34295 Montpellier, France; jorge.lopezcastroman@chu-nimes.fr; 3 Psychiatric Clinic, Faculty of Medicine, Institute of Clinical Medicine, Vilnius University, LT-03101 Vilnius, Lithuania; 4 Laboratory of Preclinical Drug Investigation, Institute of Cardiology, Lithuanian University of Health Sciences, LT-50161 Kaunas, Lithuania; martinkutelaura@gmail.com; 5 Department of Adult Psychiatry, Nimes University Hospital, 44307 Nimes, France; 6 Centro de Investigación Biomédica en Red de Salud Mental (CIBERSAM), 28001 Madrid, Spain

**Keywords:** psilocybin, suicidality, suicidal behaviors, psychedelics, psychiatry, serotonin, inflammation, oxidative stress, pharmacology, treatment

## Abstract

The available interventions for people who are at risk of suicide have limited efficacy. Recently, research on new mental health treatments has started to consider psychedelic compounds, particularly psilocybin, a molecule with a few thousand years of history of use in human societies. The possible effects of psilocybin on suicidal ideation and behaviors have not been specifically studied yet; however, the current knowledge on the suicidal process and the available data on es/ketamine suggest that psylocibin could be used to modulate the thoughts and behavioral patterns in individuals who are at risk of suicidal behaviors. Here, we summarize the available evidence on the possible mechanisms underlying psilocybin positive effects on suicide risk. Major pathways related to suicidal behaviors that might be modulated by psylocibin include serotonin receptors. Specifically, psylocibin directly stimulates the serotonin 2A receptor (5HT_2A_), targeting the inflammatory and oxidative stress pathways and leading to a rapid increase in brain plasticity and inflammation suppression and increases in cognitive flexibility, spirituality, and empathy. We also present preliminary epidemiological data and provide a rationale for studying psilocybin in individuals with suicidal ideation or who are at risk of suicidal behaviors. This review presents a framework to understand the basis for psilocybin use in individuals who are at risk of suicidal behaviors and calls for clinical studies.

## 1. Introduction

In recent decades, research on psychedelic treatments for mental disorders has gained new interest [1]. Psilocybin is one of the most studied psychedelic substances and has been associated with the sustained remission of depression for weeks, and in some patients, for years [2]. Based on these findings, the United States Food and Drug Administration defined psilocybin as a “breakthrough therapy” for treatment-resistant depression [1]. Studies on psilocybin have mainly been focused on treatment-resistant depression, addiction, eating disorders, and end of life anxiety in patients with cancer [2,3,4]. However, on the basis of its mechanisms of action, it might be useful to determine the use of psilocybin to prevent suicidal behaviors (SB) (suicide, suicide attempt (SA)) and to decrease suicidal ideation (SI) [5].

Despite its entry in the 5th edition of the *Diagnostic and Statistical Manual* (DSM5) as a putative disorder, SB are generally considered as a symptom or a consequence of a concomitant psychiatric disorder [6], most frequently major depressive disorder. Consequently, in clinical settings, antidepressants and psychotherapy remain the two main strategies to prevent SB and to reduce SI. However, classic antidepressants are not very effective in suicidal patients. Indeed, patients with depression and with current SI and/or past SA (i.e., suicidal patients) tend to respond less well to classic antidepressant treatments [7,8]. Moreover, some patients (~10% according to different studies) experience treatment-emergent SI or treatment-worsening SI, especially young (18 to 24-year-old) adults [9]. In addition, in some patients with SI (10 to 20% according to different studies), SI does not decrease despite the improvement of depressive symptomatology following treatment [10,11]. Importantly, the full clinical response to classic antidepressants is generally observed after at least two weeks, and the unfavorable side-effect profile frequently results in premature or abrupt discontinuations [12,13]. Similarly, the response delay to psychotherapy is an issue in patients with SB and SI despite the recent emergence of promising specific regimens [14]. Moreover, the high price, low accessibility, and requirement of a sustained commitment to psychotherapy can be overwhelming for patients who are experiencing a suicidal crisis [15]. SB and SI are a major public health problem: 800,000 suicides occur worldwide each year, SA are approximately 20 to 30 times more frequent, and 45 to 70% of patients with psychiatric disorders experience SI [16]. Therefore, it is crucial to find rapidly effective drugs to decrease SI and to prevent SB. New treatments are emerging (i.e., ketamine and esketamine) and have shown promising results [17,18]. However, not all patients respond to these treatments. In addition, multiple administrations are required, and these drugs most often must be taken in combination with a classic antidepressant after their intake. Psilocybin could be an interesting alternative because it (i) acts on mechanisms that are implicated in SB and SI physiopathology [5]; (ii) could produce results with just one administration [4]; (iii) acts rapidly [19]; (iv) may not require co-therapy with a classic antidepressant after intake [20]; (v) has demonstrated sustained long-term efficacy (up to 6 months) [2]; and (vi) does not present any risk of addiction or discontinuation syndrome [2]. For patients taking an antidepressant therapy, a wash-out period of at least 7 days will be needed for psilocybin intake. Thus, a progressive discontinuation of an antidepressant followed by a wash out period of at least 7 days may require strict medical supervision during hospitalization to prevent suicidal risk.

Although psilocybin and other psychedelic compounds have never been specifically tested in patients with a history of SI or SA, some previous studies reported their effects on SI and SB. For instance, the use of psilocybin and other psychedelics (e.g., lysergic acid diethylamide, LSD) has been linked to lower odds of past SB and SI in a large American epidemiological study (190,000 people), which is unlike other illicit drugs (e.g., heroin) [21]. A recent systematic review investigated the effects [22] that psychedelics used in non-clinical (e.g., recreational, mystical) and in clinical contexts have on suicidality. The results were contradictory. Indeed, some studies found an increase in suicidality, while others found a decrease or no effect. However, the authors of the review highlighted that these studies had some biases (e.g., not adjusted for potential cofounders, such as use of other drugs) and small sample sizes (many were case reports). Furthermore, it seems logical that without adequate medical supervision and careful patient selection (e.g., exclusion of patients with schizophrenia), psychedelics can have deleterious effects. On the other hand, the authors found that in more recent clinical trials, classic psychedelics, including psilocybin, showed promising results for rapidly reducing SI. Importantly, in these recent clinical trials, no suicide-related events were reported. This confirms that when used correctly (e.g., strict selection criteria, under medical supervision, taking into account all potential cofounders) these substances could have beneficial effects in patients with SB. Interestingly, two recent studies on ayahuasca (a serotoninergic agonist like psilocybin) demonstrated that this psychedelic drug decreases SI in patients with major depressive disorder and with treatment-resistant depression [23,24]. Moreover, a recent open-label study found a significant decrease of demoralization in old long-term AIDS survivors of up to 3 months after psilocybin administration [25]. In another study in patients with advanced cancer (an important risk factor of SI/SB), psilocybin-assisted psychotherapy was associated with a significant decrease of SI 8 h after the session and up to 6.5 months after treatment [19]. Similarly, an open-label study reported a significant SI reduction in patients with treatment-resistant depression 1–2 weeks after psilocybin administration [20]. Lastly, a recent randomized controlled trial in patients with major depressive episode found that psilocybin significantly decreased SI up to 8 weeks after treatment [26]. Although the effects of psilocybin on suicidality (i.e., suicide, SA, and SI) have never been specifically studied (except SI reduction in few trials), these preliminary data concerning psilocybin activity on the pathways that are impaired in SB suggest that this psychedelic drug could be useful to decrease SI and to prevent SB.

The aims of this review were (i) to describe the pharmacological properties of psilocybin and its action on systems that are known to be impaired in suicidal patients (serotoninergic system, neurotrophic factors, and inflammatory and oxidative systems) and (ii) to discuss how the psychological effects of psilocybin could explain the putative reduction of SI and SA.

## 2. Suicidal Behaviors Need Specific Therapeutics

As ~90% of people who die by cause of suicide have a psychiatric disorder (mainly depression), SB and SI are still often considered to be a consequence or a symptom of another psychiatric disorder rather than a disorder in its own [27]. Consequently, non-specific treatments are used to prevent/treat SB/SI. For example, it is generally thought that targeting the depression symptomatology will also decrease the suicide risk, which is mostly true, although not without caveats. Furthermore, as suicidal patients are almost systematically excluded from clinical trials, the treatments that have been proposed have never been tested (e.g., efficacy, tolerability, safety) in this specific patient population [28,29]. Understandably, clinicians and researchers are afraid to test medications on suicidal patients for various reasons (e.g., participant safety, decisional capacity), but it is now possible to conduct a safe protocol within these patients, as seen in those conducted with ketamine and esketamine [28,29]. Furthermore, growing evidence suggests that although SB and SI are certainly associated with other disorders, they have their own physiopathology [30]. This is stressed by the entry of SB in the DSM5, which denotes it as a pathology that should be studied further. This suggest that SI/SB must be studied independently and in addition to depression. On the one hand, suicidal patients do not respond as well to antidepressant treatment (e.g., persistence of SI despite remission of depression [11]), and on the other hand, it seems that the physiopathology of depression with and without SI is different.

To support this, several recent studies suggest that patients with depression and SB/SI could represent a specific group that is different from patients without SB/SI. Depression (the most frequent accompanying diagnosis in patients with SB/SI) and SB/SI are two separate dimensions that can often overlap [31]. A recent study described two trajectory types for SI and for depressive symptoms, with independent class membership for the two outcomes [32]. In addition, the latent variable structures (i.e., the factorial structures of scales used to measure psychopathology) are significantly different in patients with depression according to the presence/absence of SI [33]. Furthermore, clinical characteristics (e.g., higher hopelessness, psychological pain and anxiety and more sleep disturbances) are more severe, and the course of the depressive symptoms is different in suicidal patients with depression (unipolar or bipolar disorder) compared to non-suicidal patients with depression [10,11,34,35]. Laboratory/imaging parameters are also different between suicidal and non-suicidal patients [30,36]. Indeed, for instance, it has been found that depressed patients with lifetime SA present lower serotonin transporter binding in the midbrain than a depressed subject without history of SA and healthy controls [37]). In the same vein, neuro-imaging studies in patients with psychiatric disorders showed that some brain areas (e.g., prefrontal cortex, amygdala) present different activation profiles during social exclusion or during decision-making process with emotional feedback in the function of whether there is a presence of a history of SA or not [38,39]. Basal cortisol levels and interleukin (IL)-2 levels are lower in suicidal patients than they are in patients with depression [40].

These findings and the absence/limited response to classic antidepressants reinforce the hypothesis that suicidal patients represent a specific group and that they need specific therapeutics to specifically target the systems that are involved in SB/SI.

## 3. Psilocybin Pharmacological Properties

Psilocybin, or 4-phosphoryloxy-*N*,*N*-dimethyltryptamine, is an indoleamine. It is the main psychoactive compound of *Psilocybe* mushrooms and is one of the so-called “classic” serotonergic hallucinogens [41]. Psilocybin has been integrated in cultural, religious, and spiritual practices for thousands of years before it attracted the interest of the scientific community. In 1958, Albert Hofmann and his colleagues at Sandoz Laboratories identified and synthesized psilocybin and its most prominent active metabolite psilocin (4-hydroxy-*N*,*N*-dimethyltryptamine) [42,43]. Later, psilocybin was marketed under the name of Indocybin^®^ as a promising agent for psychiatric disorders.

### 3.1. Pharmacokinetics

Even if psilocybin is not a novel therapeutic, a brief reminder on its pharmacokinetics is needed in order to better understand this molecule. The chemical structure of psilocybin as well as its other parameters suggest that it cannot freely cross the blood–brain barrier, unlike its metabolite psilocin, which is more lipophilic than the parent drug [44,45]. Therefore, psilocybin should be considered as a pro-drug, and psilocin should be considered as the active metabolite [46]. After oral administration, psilocybin is quickly converted to psilocin in the acidic environment of the stomach or by the action of alkaline phosphatases, probably by luminal and first-pass dephosphorylation [45,46].

In humans, psilocin is detected in the plasma 20–40 min after oral administration [47], and the plasma concentration reaches a peak (mean concentration: 8.2 ± 2.8 ng/mL) at 80–105 min post-administration [47,48]. The estimated normalized bioavailability of psilocybin is ~50% [48]. Unsurprisingly, the intravenous administration of psilocybin has been associated with faster mean maximum plasma level peaks of psilocin: 12.9 ± 5.6 ng/mL at 1.9 ± 1.0 min post-injection.

A more recent open-label study [49] analyzed the pharmacokinetics and safety profile of psilocybin (sequential, escalating oral doses of 0.3, 0.45, and 0.6 mg/kg) in 12 healthy adults. Psilocybin was not found in plasma or urine, and the renal clearance of intact psilocin accounted for less than 2% of the total clearance. This confirmed that psilocybin is dephosphorylated to psilocin. Although doses of 0.6 mg/kg are higher than the dose that is generally used in clinical trial settings, no serious side effect was reported in the month following administration [50]. The differences between the oral and intravenous administration of psilocybin are the speed of onset and the intensity of the subjective effects. After oral administration, the first effects are observed after approximately 40 min, and they last 4–6 h [51]. Conversely, after the intravenous administration of 2 mg psilocybin, effects in healthy adults peaked after 4 min and diminished after 45–60 min [52].

Studies in rats have shown that the half-life of psilocin in plasma is 2.5 h after oral ingestion and is 1.23 h after the intravenous administration of psilocybin [53]. Most of the drug is excreted 3 h after ingestion and is eliminated from the body within 24 h [54]. Psilocin and its metabolites are mainly excreted in the urine (approximately 65% of the administered dose in 24 h), followed by elimination in bile and feces (15–20%). Most of these metabolites are excreted within the first 8 h, but up to 20% are retained for longer, and significant quantities are still found in urine at day 7 post-administration [55].

### 3.2. Pharmacodynamics

Classic psychedelics differ between each other in terms of receptor binding specificity. Preclinical studies have shown that psychedelics, including psilocybin, exert their hallucinogenic effects through serotonin receptor activation in the cortical and subcortical structures [56]. Studies in rats showed the typical signs of stimulation of the 5-hydroxytryptamine 2A (5-HT_2A_) receptors, such as head twitching and wet-dog shakes, after the injection of psilocybin. These behaviors could be blocked or significantly reduced by the pharmacologic inactivation of the 5-HT_2A_ receptors [57].

Psilocybin mainly interacts with serotonergic system components, such as the 5-HT_1A_, 5-HT_2A_, 5-HT_2B_, and 5-HT_2C_ receptors [53]. This is relevant for SB because SB have been linked to alterations in the serotonergic system [58,59]. Pre-treatment with the 5-HT_2A_ receptor antagonist ketanserin blocks most of the psychedelic effects of psilocybin, suggesting that they are mainly mediated through the activation of postsynaptic the 5-HT_2A_ receptors [56,60,61,62]. The agonism of the 5-HT_2A_ receptor has been linked to increased memory formation and learning as well as to the contraction of the bronchial and gastric smooth muscles as well as the cardiovascular and gastrointestinal anti-inflammatory effects and the release of certain hormones [45]. The plasma levels of psilocybin directly correlate with neocortical 5-HT_2A_ stimulation and the subjective evaluation of its psychoactive effects [63]. In studies in healthy participants, positron emission tomography imaging showed that psilocybin increases the absolute metabolic rate of glucose in the frontal cortical regions as well as in the striatal and limbic subcortical structures [64,65,66].

Although the binding to the 5-HT_2A_ receptors explains most of the effects of psilocybin, interactions with other pre- and post-synaptic 5-HT subtypes might also contribute. However, the available data are limited. It has been shown that psilocin binds to many different serotonin receptors [67,68]. Furthermore, 5-HT_2A_ receptor activation by psilocybin could modulate striatal dopamine release because psilocin increases extracellular dopamine levels in the mesoaccumbens pathway in rats that are awake [69]. This could help to ameliorate the reward deficits in suicidal patients [70] in whom the reward-related pathways are impaired. For example, individuals who attempt suicide show prefrontal alterations during reward-based learning and decision making with emotional feedback [39]. Moreover, SB have been associated with impaired reward-based learning, and this might undermine the search for alternative solutions [71]. A recent electroencephalography study showed that suicidal individuals display specific deficits in reward anticipation [72]. In addition, increased dopamine levels enhance pleasure. For example, the hedonic experience of music is increased by levodopa (dopamine precursor) and is decreased by risperidone (dopamine receptor antagonist) [73]. Similarly, another experimental study showed that increasing dopamine levels enhances motor vigor, a proxy to effort allocation for high rewards [74]. A psilocybin-mediated dopamine level increase could have similar effects that would be especially beneficial in suicidal individuals.

## 4. Neuroplastic Changes in Neurons and Synapses

Psilocybin-mediated neuroplastic changes are mediated through the brain-derived neurotrophic factor (BDNF) [75] that is implicated in brain neurogenesis, neuroplasticity, and regeneration. By stimulating the 5-HT_2A_ receptors on large glutamatergic pyramidal cells in the deep cortical layers (V and VI) projecting to the layer V pyramidal neurons, psilocybin increases the extracellular glutamate levels in the pre-frontal cortex (PFC) [76]. Glutamate activates the alpha-amino-3-hydroxy-5-methylisoxazol-4-propionate (AMPA) and N-methyl-D-aspartate (NMDA) receptors on the cortical pyramidal neurons, resulting in increased PFC neuroplasticity via BDNF increase and other mechanisms [76]. A transient state of high neuroplasticity can already be observed after a single dose of psilocybin, which could translate into long-lasting synaptic changes [77]. This hypothesis is supported by the finding that AMPA receptors play a major role in neural network formation during development [78]. Besides its action on BDNF, psilocybin rapidly increases the expression of genes related to neuroplasticity (e.g., *c-Fos*, *Junb*, *Dusp1*, *Iκβ-α*) in the PFC and to a lower extent, in the hippocampus of rats [79]. Another recent in vivo study in mice demonstrated that psilocybin increases the density and strength of neuronal connections by about 10% in the medial frontal cortex [80]. This led to an increase of excitatory neurotransmission. The growth of dendritic spines was observed 24 h after psilocybin intake and persisted up to 1 month. Psilocybin might also stimulate neuroplasticity by activating the mammalian target of rapamycin (mTOR). Indeed, psilocybin promotes neuritogenesis, resulting in increased dendritic arbor complexity (higher number of dendritic spines and higher connections). However, this effect is blocked by treatment with rapamycin (an mTOR inhibitor) [81]. In in vivo studies, the stimulation that is achieved in the molecular and neuronal pathways related to neuroplasticity after treatment is psychedelics is accompanied by an increase in learning behavior [82]. Furthermore, in the acute state, psilocybin intake leads to a global decrease in functional network integrity but higher connectivity between networks. Magnetoencephalography and electroencephalography studies found that psilocybin causes a major loss of rhythmical activity, resulting in a state of extreme desynchronization or enhanced entropy in the acute state as well [2]. By escaping from its usual way of working and due to the global increase in connectivity, the brain might create new behavioral and thought patterns. Indeed, the cortical disintegration of the default mode network has been related to changes in thoughts and behavior [77]. The acute desynchronization and the increase in neuroplasticity in the long-term are complementary. Indeed, both are needed to stimulate the creation of news networks. Consequently, psilocybin could improve functional integration and decrease negative thinking and rumination.

These effects could be beneficial in patients who are at a high risk of SB because SB have been associated with neuroplasticity dysfunction (e.g., low BDNF levels) [40,59]. Indeed, current evidence suggests that brain neuroplasticity is altered in most SB-associated psychiatric disorders [83]. Furthermore, postmortem studies showed that BDNF levels are significantly lower in different brain regions, especially in the hippocampus and PFC, in patients who died from suicide [84]. Epigenetic modifications that alter BDNF expression have also been detected in patients who died by suicide [85]. In agreement, a recent meta-analysis found that plasma BDNF levels are significantly lower in patients with depression with than in patients without a history of SA [84]. Moreover, psilocybin-mediated neuroplasticity seems to be linked to mTOR induction. This is particularly interesting because the expression of the mTOR protein and its related genes has been strongly linked to death by suicide and SB. Indeed, mTOR expression has been found to be lower in patients who died from suicide and with SB [86,87]. Thus, by promoting mTOR expression, psilocybin could compensate for its decrease observed in suicidal patients. Finally, suicide has been associated with lower cortical thickness, a decreased number of dendritic spines, and the atrophy of neurons in the PFC [88,89]. By increasing the number of dendritic spines and by promoting neuritogenesis, psilocybin could correct this phenotype. In view of these elements, it seems that the psilocybin modulation of neuroplasticity could support its putative anti-suicidal effect.

## 5. Anti-Inflammatory Effects of Psilocybin

*Psilocybe* mushrooms have a specific anti-inflammatory effect. It has been reported that extracts of these mushrooms inhibit the lipopolysaccharide-induced production of the pro-inflammatory cytokine tumor necrosis factor α (TNF-α) and IL-1β and decrease the concentration of IL-6 and cyclooxygenase 2 (COX-2) in human U937 macrophage cells [90]. Moreover, the mushroom *Psilocybe cubensis* protects cardiomyocytes against TNF-α-induced injury and cell death [91]. These effects are exerted by mimicking the action of serotonin on 5-HT_2A_ receptors [92].

Increasing evidence supports the notion that serotonin modulates inflammation in the brain. In psychiatry, the role of inflammation-related kynurenine pathway alterations in mood disorders has been extensively studied [93,94,95]. Indoleamine 2,3-dioxygenase is the most important enzyme that shifts the metabolism of tryptophan to serotonin in the kynurenine pathway. One of its main inductors is TNF-α [96,97]. It has been shown that the pharmacological agonists of the 5-HT_2A_ receptors block the pro-inflammatory effects of TNF in smooth muscle vascular cells [98] and that they a potency that exceeds that of all of the current drugs or small therapeutic molecules. However, these effects are restricted to 5-HT_2A_ receptors because 5-HT_2B_ and 5-HT_2C_ receptor-selective agonists cannot suppress TNFα-induced inflammation, further supporting the major role of 5-HT_2A_ binding.

Indirect evidence has also shown that single-nucleotide genetic polymorphisms in the 5-HT_2A_ receptor gene are associated with rheumatoid arthritis [99], a disease that is linked to increased TNF-α levels, and are responsive to TNF-α antagonists. Interestingly, mirtazapine, a potent 5-HT_2A_ antagonist, increases TNF-α levels [100,101]. Furthermore, a retrospective analysis reported a 45-fold excess rate of joint disorder complaints in patients treated with 5-HT_2A_-blocking antidepressants, such as mianserin, nefazodone, and mirtazapine, compared to patients treated with selective serotonin reabsorption inhibitors that indirectly stimulate 5-HT_2A_ receptors [102].

Interestingly, mirtazapine is among the least effective antidepressants that can be used for the prevention of SA and death by suicide in patients with major depressive disorder [103]. Different mechanisms could contribute to this. First, by blocking the 5HT_2A_ receptors, mirtazapine could impair neuronal plasticity. Second, mirtazapine is not a serotonin reuptake inhibitor and is very unlikely to induce serotonin toxicity compared to many other antidepressants. It modestly increases serotonin levels only indirectly through alpha 2 receptor antagonism [104]. This may be sufficient to treat patients with depression, but not enough for suicidal patients. Nevertheless, the most likely explanation is probably linked to 5HT_2A_ impairment [101] because suicide has been associated with increased levels of pro-inflammatory cytokines in the brain [105].

The mechanisms underlying the anti-inflammatory actions of 5-HT_2A_ agonists are not entirely clear [106]. Studies on psilocybin and LSD (which is closely related to psilocybin and also activates the 5HT_2A_ receptors_)_ found significant acute effects on circulating steroids, especially glucocorticoids [107,108]. As glucocorticoids have major anti-inflammatory properties, their rapid increase might immediately suppress inflammation, reducing chronic inflammation, as commonly observed in patients with depression, especially those who are at an increased suicide risk. Indeed, in patients with depression, the hypothalamic–pituitary–adrenal axis is deregulated, the glucocorticoid response to stress is flattened, morning cortisol concentrations are low, and glucocorticoid receptor resistance is observed. Moreover, SA history and SI have been specifically linked to decreased cortisol response to stress [109,110]. Therefore, a psilocybin-induced significant release of anti-inflammatory cortisol, corticosterone, cortisone, and 11-dehydrocorticosterone could activate an anti-inflammatory response (similar to how insulin activates insulin receptors in insulin-resistance syndrome) and could reduce the levels of pro-inflammatory cytokines, such as TNFα and IL-6 [107].

It is not fully understood whether these anti-inflammatory effects are generated upon the central or peripheral activation of the 5-HT_2A_ receptors. Some studies suggest that it is the subjective intensity of the subjective experience that produces the therapeutic effects [111,112]. However, 5-HT_2A_ receptor (mRNA and protein) expression has also been detected in many peripheral immune-related tissues (e.g., spleen, thymus, and circulating lymphocytes) [113] and in innate and adaptive immune response cells (e.g., eosinophils [114] human peripheral blood mononuclear cells [115], and T cells [116]). One may wonder whether a psilocybin-like molecule that does not cross the BBB and does not have subjective psychedelic effects may still be effective [117,118]. One rodent study found that psilocybin and LSD have a persistent antidepressant-like effect, but not ketamine. As it is impossible to measure the subjective effects in rodents (only head-twitch behaviors can be measured, but this was not done in this study), the authors hypothesized that subjective experience may not be necessary for the therapeutic effects of these molecules [119].

The anti-inflammatory effects of psilocybin could be useful in suicidal behaviours, as patients with history of SA and SI have increased levels of inflammatory markers [40,93], psilocybin might rapidly reduce this chronic low-grade inflammation, restoring the brain plasticity capacity. Then, psilocin strongly binds to the 5-HT_2A_ receptors, the expression of which is increased in postmortem brain samples from patients with depression and suicidal tendencies [120,121,122]. The alterations in the 5HT_2A_ receptors in the brain [123] and in the peripheral tissues (platelets) [124] have been associated with suicidality. Moreover, two polymorphisms of this receptor have been linked to SA [125]. It has been proposed that 5HT_2A_ receptor upregulation is a compensatory mechanism that can decrease the availability of serotonin or the increased demand of 5-HT_2A_ function [126,127]. As classic antidepressants decrease 5-HT_2A_ density, it has been suggested that 5-HT_2A_ receptor downregulation may be the underlying mechanism of their effect [128]. However, the effect of traditional antidepressants is generally observed after few weeks of treatment, whereas psilocybin induces this downregulation rapidly. This might explain the rapid antidepressant effect observed in the clinical studies that have been conducted on psilocybin [129]. Therefore, 5-HT_2A_ and 5-HT_1A_ signaling normalization might help to explain the possible antidepressant and anti-suicidal effects of psilocybin [130].

## 6. Antioxidant Effects of Psilocybin

It is known that indole ring-containing molecules, including psilocybin, have antioxidant effects [131,132,133]. Mushrooms are rich in antioxidants, such as ergothioneine and glutathione [134]. However, specific data on the antioxidant effects of *Psilocybe* mushrooms are scarce. One study reported that *Psilocybe natalensis* has a potent antioxidant effect [135]. Studies on other similar molecules support this putative antioxidant effect. For instance, dimethyltryptamine (DMT), which is an endogenous neurotransmitter, displays potent protective effects against hypoxia by acting on sigma 1 receptors [136]. It has been hypothesized that the main purpose of endogenous DMT is to protect the brain in case of hypoxia [137]. Moreover, a study demonstrated that the closely related 5-methoxy-N, N-DMT (5MeO-DMT) rapidly decreases IL-6 levels and increases cortisol levels [138]. As such changes have been associated with reduced suicide risk [139,140], these effects might be implicated in the anti-suicidal effects of these molecules.

Indirect evidence also supports these observations. The activation of 5-HT_2A_ inhibits the activity of the inducible nitric oxide synthase in C6 glioma cells, reducing oxidative stress [141]. Selective serotonin reabsorption inhibitors also suppress oxidative stress [142,143,144]. Although the underlying mechanism is not clear, the modulation of 5-HT_2A_ receptor and brain steroid expression has been implicated [145].

Similar to inflammation, oxidative stress also has been associated with SB [146]. A recent meta-analysis found an association between SA history and increased nitro-oxidative stress [147]. Many abnormalities in oxidative stress systems have been implicated in SB pathophysiology [148]. Moreover, it has been suggested that classic antidepressants reduce depressive symptomatology and SI by improving oxidative stress and antioxidant function [149].

## 7. Neuropsychological Aspects

As described previously, psilocybin alters the default mode network connectivity, and this might enhance cognitive flexibility [1]. This rapid increase of cognitive flexibility could mediate the switch from avoidance to acceptance thought and behavioral patterns. It is thought that cognitive flexibility and acceptance are increased in response to individual experience. Interestingly, this change might be maintained for a long time after psilocybin intake [150]. Such remodeling might be particularly important in suicidal patients because they present altered decision making, reduced cognitive flexibility, and poor problem-solving ability [30]. In addition, SB could be considered as the most extreme expression of avoidance, and acceptance therapy is effective in patients with SI [14].

Increased empathy is another psychological change that has been associated with psilocybin administration [77]. The theory of “ego dissolution” (i.e., a disrupted sense of self [151] following psilocybin intake) suggests that the experience caused by psilocybin allows patients to be more open to their social surroundings [77]. This may lead to an increase in connectedness with the surrounding environment and people. This effect could be important in patients who are at an increased risk of suicide because they often present thwarted feelings as though they belong [152]. Therefore, one can speculate that by promoting connectedness, psilocybin might help to prevent SB. Moreover, it has been shown that psilocybin significantly improves emotional face recognition [153] and reduces feelings of social exclusion [154]. Patients with a history of SA are particularly sensitive to social exclusion [38]. Psilocybin might reverse this excessive feeling of not-belonging.

SB has also been associated with reduced specific autobiographical memories [40]. A meta-analysis reported that long-term memory is significantly impaired in patients with history of SA compared to healthy controls as well as compared to psychiatric patients without a history of SA. Moreover, it has been observed that autobiographical memory is less specific in patients with SA history [155]. It has been hypothesized that the system that is involved in thinking about the future overlap with the one that is implicated in episodic memory. Consequently, the capacity to solve problems and to find solutions could also be impaired [155]. On the other hand, psilocybin administration enhances autobiographical recollection by stimulating the recall/re-experiencing of autobiographical memories and by accentuating the vividness of memories during its acute effects [156,157]. A functional magnetic resonance imaging study demonstrated the greater activation of the bilateral auditory cortex, somatosensory cortex, superior parietal cortex, and occipital pole following the administration of psilocybin compared to a placebo [156]. This could explain the vividness of memories upon psilocybin intake. This visual and sensorial activation and the reports of more visual and vivid recollections after psilocybin administration suggest that psilocybin stimulates the neural processes underlying autobiographical recall. This effect is amplified by music during the session. Indeed, music increases the visual imagery that is involved in autobiographical memory [150]. Interestingly, recent studies have shown that emotional breakthrough (EB) also contributes to the increased well-being after psilocybin intake [158,159]. EB overlaps with the psychoanalytic notion of catharsis and is influenced by the context. Greater EB during the psychedelic experience has been linked to greater well-being afterwards [159]. It could be hypothesized that EB occurs during the autobiographical recall induced by psilocybin and “liberates” patients from the negative emotions that are linked to their memories. This could be useful in suicidal patients who often have history of childhood trauma, other negative life events, and/or biased memories. Indeed, the recall of their trauma or negative life events and/or a biased memory accompanied by the effects of psilocybin discussed here could help them to overcome these memories by liberating them from the negative emotions that are associated with them. Thus, it is primordial for suicidal patients to be briefed before the session and to be supported throughout it by a professional.

**Figure 1:**
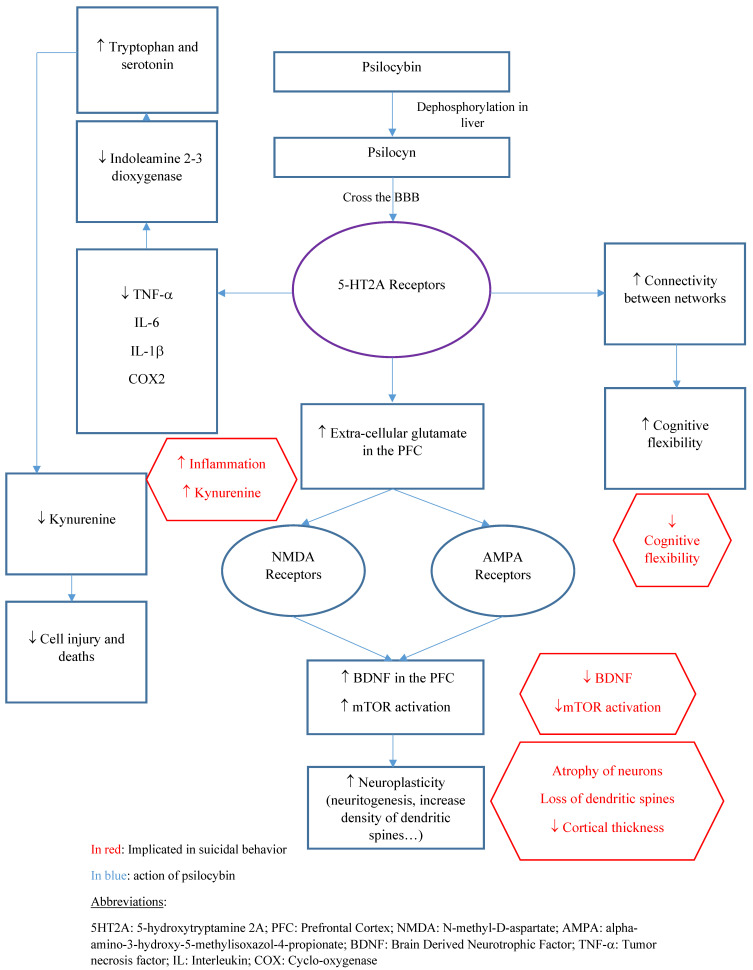
Main mechanisms of action of psilocybin that could be useful to prevent SB.

Finally, psilocybin administration has been associated with “mystical” or “quantum change” experiences. This last term also takes into account the long-term changes that are associated with such experiences. A “quantum change” experience can be defined as a “sudden, distinctive, benevolent and profoundly meaningful experience resulting in personal transformation that affects a broad range of emotions, cognitions and behaviors” [160]. Such experiences (e.g., mystical, quantum change, religious) might lead to sustained behavioral changes. For instance, the total scores of the Mystical Experience Questionnaire following psilocybin administration positively predict psilocybin-related changes in behavior and well-being [1]. Moreover, previous therapeutic trials found a positive association between the magnitude of mystical experiences and sustained positive outcomes (e.g., well-being, positive attitude, and mood) [161]. Thus, psilocybin may enhance spirituality, which is also associated with a reduced suicide risk [162]. Figure 1 summarizes the main mechanism of possible psilocybin antisuicidal action.

## 8. Risks

As is the case with any medical intervention, the safety of an intervention must be confirmed before assessing its efficacy. Psilocybin has a favorable physiological safety profile [108]. Although it can raise the heart rate and blood pressure, cardiovascular events and deaths have never been recorded following psilocybin administration. Moreover, there could be yet unknown risks from psilocybin acting in peripheral tissues, as 5HT_2A_ and other receptors are also expressed there. Concerning overdose, due to its pharmacodynamic profile, psilocybin overdose and dependence are also unlikely because tolerance builds up quickly due to the rapid receptor desensitization [129,163].

However, the psychological risks that are associated with psilocybin should not be neglected [164]. The patient must be psychoeducated about the reality-altering effects of this drug that can be disturbing for many and traumatizing for some. Although some people may seek these mind-altering effects, patients with psychiatric diseases generally just want to feel better. Therefore, hallucinations could be considered as a side effect that can be minimized with psychological preparation and that can be accepted if the anti-inflammatory properties of psilocybin also are taken into account. In people with a personal or family history of psychosis, the risk-benefit ratio might be less favorable.

After treatment with psilocybin, follow-up with a trained specialist is of the utmost importance to integrate the experience. Indeed, the molecule produces reality-altering effects and can also bring up various traumatic memories, inducing a fear response. For instance, case reports have described post-traumatic stress disorder following a challenging psilocybin experience [165]. Moreover, the theoretical possibility of a rare condition, called hallucinogen persisting perception disorder [166,167], should also be considered. Although its existence is discussed and it seems to be responsive to antipsychotic or benzodiazepine, it might cause major psychological suffering [168].

Clinicians and researchers may be afraid to test this drug in suicidal patients because many think that without good monitoring, psilocybin can be quite stressful and may lead to a suicidal act. These negative responses could be favored by psychiatric comorbidities. However, safe clinical trials in these patients can be conducted using an adapted protocol (e.g., staying with the patient throughout the session, confirming the patient’s family and social support, preparing emergency plans with the patient, implementing strict clinical monitoring) [28,29,169].

## 9. Conclusions

In this review, we summarized the existing data on the mechanisms that might underlie the putative anti-suicidal effects of psilocybin, a naturally occurring compound. This molecule acts by binding to the 5-HT_2A_ receptors, resulting in a rapid decrease of inflammation and oxidative stress and neuroplasticity promotion. These effects might underlie the shift from the cognitive patterns that are frequently observed in patients with SA history and SI (e.g., cognitive rigidity, impaired decision making, and feelings of a thwarted sense of belonging) to more adaptive thoughts and behaviors (e.g., increased cognitive flexibility, spirituality, and empathy).

Despite the great deal of progress that has been made in suicide prevention, the current therapies are insufficient, and there is a large unmet care need. Psilocybin seems safe, rapidly effective, and has been used for thousands of years due to its presence in the natural world. We propose that the effects of psilocybin and ketamine should first be compared in patients some days (at least 7) after a suicidal crisis (i.e., SA and/or hospitalization for SI), regardless of the associated psychiatric disorder (i.e., depressive disorder and bipolar disorder), as ketamine is already used in clinical practice for these patients. Patients who are at risk (e.g., schizophrenia, psychotic symptoms, imminent risk of suicidal act) must be excluded, and psychotherapy (e.g., mindfulness therapy) must be offered after psilocybin intake under strict medical supervision. This first trial type could allow for the assessment and confirmation of the safety and efficacy of psilocybin before studying its effects in “active” suicidal patients (i.e., with active SI). Future clinical trials will then specifically investigate psilocybin effects on SI and SB risk as a primary outcome in order to determine the best optimal dose, clinical settings, safety, and interactions with current pharmacotherapies and psychotherapies [170].

## Data Availability

Not applicable.
